# Different gene expression of *MDM2*, *GAGE-1*, *–2* and *FHIT* in hepatocellular carcinoma and focal nodular hyperplasia

**DOI:** 10.1038/sj.bjc.6690324

**Published:** 1999-04

**Authors:** T Schlott, K Ahrens, I Ruschenburg, S Reimer, H Hartmann, M Droese

**Affiliations:** Department of Cytopathology, Division of Gastroenterology and Endocrinology, Georg-August-University, Robert-Koch-str. 40, 37075 Goettingen, Germany

**Keywords:** fine-needle aspiration biopsy, hepatocellular carcinoma, focal nodular hyperplasia, *MDM2*, *GAGE-1*, *GAGE-2*, *FHIT*

## Abstract

Overexpression and/or mutations of oncogenes, tumour suppressor genes and tumour rejection genes have been observed in several human malignancies. Their analyses might be of diagnostic importance. Therefore, malignant hepatocytes derived from hepatocellular carcinoma (HCC) tissue as well as non-malignant hepatocytes derived from focal nodular hyperplasia (FNH) were studied. Samples containing normal human hepatocytes (HC) served as controls. Cellular material was obtained by fine-needle aspiration biopsy guided by ultrasound. Cells were analysed for expression and mutation of the oncogene *MDM2*, the genes *GAGE-1*, *-2* coding for tumour-associated antigens and the candidate tumour suppressor gene *FHIT*. Different patterns of non-mutant *FHIT* transcripts including precise deletion of exons were found in 7/10 HCC, 2/10 FNH and 2/10 HC. However, expression of non-mutant *GAGE-1*, *-2* RNA was demonstrated exclusively in 6/10 HCC samples. Further genetic features specific of HCC were point mutations in a zinc-finger motif of *MDM2* (3/10 HCC samples). Neither *GAGE-1*, *-2* expression nor *MDM2* mutations were observed in the FNH samples, or in normal hepatocytes. Our findings suggest that occurrence of variable *FHIT* transcripts is not restricted to hepatic malignant tumours. In contrast, *MDM2* mutations and *GAGE-1*, *-2* expression were associated with HCC specimens. Therefore, the RT-PCR assays for *GAGE-1*, *-2* and *MDM2* might be useful adjuncts in cytodiagnosis of liver neoplasms. © 1999 Cancer Research Campaign


*British Journal of Cancer* (1999) **80**(1/2), 73–78
© 1999 Cancer Research Campaign
Article no. bjoc.1998.0324

### Different gene expression of *MDM2*, *GAGE-1*, *–2* and *FHIT* in hepatocellular carcinoma and focal nodular hyperplasia

**T Schlott[1], K Ahrens[1], I Ruschenburg[1], S Reimer[1], H Hartmann[2] and M Droese[1]**

[1]Department of Cytopathology, [2]Division of Gastroenterology and Endocrinology, Georg-August-University, Robert-Koch-str. 40, 37075 Goettingen, Germany

**Summary** Overexpression and/or mutations of oncogenes, tumour suppressor genes and tumour rejection genes have been observed in several human malignancies. Their analyses might be of diagnostic importance. Therefore, malignant hepatocytes derived from hepatocellular carcinoma (HCC) tissue as well as non-malignant hepatocytes derived from focal nodular hyperplasia (FNH) were studied. Samples containing normal human hepatocytes (HC) served as controls. Cellular material was obtained by fine-needle aspiration biopsy guided by ultrasound. Cells were analysed for expression and mutation of the oncogene *MDM2*, the genes *GAGE-1*, *-2* coding for tumour-associated antigens and the candidate tumour suppressor gene *FHIT*. Different patterns of non-mutant *FHIT* transcripts including precise deletion of exons were found in 7/10 HCC, 2/10 FNH and 2/10 HC. However, expression of non-mutant *GAGE-1*, *-2* RNA was demonstrated exclusively in 6/10 HCC samples. Further genetic features specific of HCC were point mutations in a zinc-finger motif of *MDM2* (3/10 HCC samples). Neither *GAGE-1*, *-2* expression nor *MDM2* mutations were observed in the FNH samples, or in normal hepatocytes. Our findings suggest that occurrence of variable *FHIT* transcripts is not restricted to hepatic malignant tumours. In contrast, *MDM2* mutations and *GAGE-1*, *-2* expression were associated with HCC specimens. Therefore, the RT-PCR assays for *GAGE-1*, *-2* and *MDM2* might be useful adjuncts in cytodiagnosis of liver neoplasms.

**Keywords:** fine-needle aspiration biopsy; hepatocellular carcinoma; focal nodular hyperplasia; *MDM2*; *GAGE-1*, *GAGE-2*; *FHIT*

The oncogene *MDM2* located on chromosome 12 is amplified in solid tumours (Fakharzadeh et al, 1991; Oliner et al, 1992). Overexpression of the gene is an important tumorigenic mechanism immortalizing rat fibroblasts (Finlay, 1993) and is associated with tumour progression in leukaemias (Bueso-Ramos et al, 1993). *MDM2* encodes a phosphoprotein with multiple functions. The N-terminus of MDM2 protein forms a complex with the central tumour suppressor p53, thus blocking the *trans*-activating functions of p53 (Momand et al, 1992). The central acidic domain of MDM2 interacts with the TATA-binding protein TBP (Leveillard and Wasylyk, 1997). The C-terminus of MDM2 contains three zinc-finger motifs, two of them forming a RING finger structure (Boddy et al, 1994) which binds the basal transcription factor TAFII250 (Leveillard and Wasylyk, 1997). These findings suggest an important role of MDM2 protein in regulation of cell division and transcriptional control.

So far, only a few publications are available on the *GAGE-1* and *GAGE-2* genes. Both genes are members of a family of closely related genes coding for the MAGE-GAGE-and BAGE-antigens presented by MHC class I molecules (Van der Bruggen et al, 1991; Gaugler et al, 1994; Böel et al, 1995). The GAGE-1 and GAGE-2 tumour-associated antigens are recognized by autologous cytolytic T lymphocytes (Van den Eynde et al, 1995) and are expressed in tumour entities such as ovarian, colorectal and lung carcinomas (Van den Eynde et al, 1995; Russo et al, 1996). The GAGE-1 and GAGE-2 antigens are also testis antigens because of their absence in normal tissue except for testis (Van den Eynde et al, 1995). Therefor, they are proposed as diagnostic markers.

The gene *FHIT*, a new member of the human histidine triad gene family, has been isolated recently (Ohta et al, 1996). This gene is located on the short arm of chromosome 3 in a region carrying multiple tumour suppressor genes presumably involved in cancer development (LaForgia et al, 1991; Kastury et al, 1996). Genetic studies have defined this locus as unstable. *FHIT* contains fragile sites, one of which represents the t(3;8) breakpoint observed in familial renal cell carcinoma (Cohen et al, 1979; Wang and Perkins, 1984), while the other one, designated *FRA3B*, was originally described as one of the most fragile sites of the human genome (LeBeau and Rowley, 1984). *FHIT* is subjected to genetic alterations such as homozygous deletions or splicing abnormalities. Many samples obtained from malignant tumours of the oesophagus, stomach, colon, cervix, breast, and head and neck revealed aberrant *FHIT* transcripts containing exon deletions and insertions (Negrini et al, 1996; Sozzi et al, 1996; Thiagalingam et al, 1996; Virgilio et al, 1996; Gemma et al, 1997; Hendricks et al, 1997). Furthermore, in small cell lung carcinomas and non-small cell lung cancers, microsatellite polymorphism analyses, loss of hybridization (LOH) analyses and hybridization experiments revealed characteristic alterations within *FHIT* (Fong et al, 1997; Sozzi et al, 1996, 1997) supposed to represent initial events of lung cancer development (Sozzi et al, 1997).

*Received 14 July 1998*
*Revised 30 October 1998*
*Accepted 11 November 1998*

*Correspondence to:* T Schlott

This study is a part of the doctoral dissertation of cand. med. K Ahrens

The aim of the present study was to search for genetic alterations that might serve as markers in cytologically based diagnosis of malignant hepatic tumours. The data published so far regarding *MDM2*, *GAGE-1*, *-2* and *FHIT* suggest that analysis of the latter might contribute to the differentiation of malignant hepatocytes [i.e. derived from hepatocellular carcinoma (HCC)] from benign proliferating hepatocytes [i.e. focal nodular hyperplasia (FNH)] and normal hepatocytes respectively. Therefore, a combined expression assay for *FHIT*, *GAGE-1*, *-2*, and *MDM2* in cells obtained by fine-needle aspiration was used and these genetic data were correlated with clinicopathological features.

### MATERIALS AND METHODS

### Sample collection

Slides examined were obtained by ultrasound-guided fine-needle aspiration from ten patients with HCC (five women, five men) and ten patients with FNH (eight women, two men). In all cases, cytodiagnosis was subsequently confirmed by histological and/or clinical findings. The slides were routinely stained according to May–Grünwald–Giemsa and stored up to 3 years. Additionally, ten controls (hepatocyte cultures, fetal hepatocytes, specimens from liver autopsy) were analysed. Two cell lines isolated from fresh HCC samples described in the previous study of Schlott et al (1997) were used for establishing the tests.

### RNA isolation and cDNA amplification

Glass slide smears were kept in xylene for 2 days to remove cover slips. The cells were scraped into sterile Eppendorf microfuge tubes with razor-blades and washed once with xylene and twice with ethanol (96%, v/v) to remove any traces of xylene. Cells and cellular debris were harvested by centrifugation (500 rpm at 4°C) and dried in a speed vacuum concentrator (Eppendorf, Hamburg, Germany). RNA was isolated from malignant and non-malignant hepatocytes with the RNeasy Total RNA Kit (QIAGEN, Hilden, Germany) according to the manufacturer's instructions. RNA was eluted from the affinity columns and concentrated in a final volume of 5 µl water by vacuum centrifugation. To inhibit degradation of RNA, plastic ware and solutions were treated with 0.1% (w/v) DEPC (Sigma, Munich, Germany). Oligonucleotide primers purified by high-pressure liquid chromatography were supplied by MWG Biotech (Munich, Germany). Amplification of RNA transcripts was performed with a GeneAmp RNA-PCR Kit according to the manufacturer's prescriptions (Perkin-Elmer, Weiterstadt, Germany). Polymerase chain reaction (PCR) was performed on a DNA thermal cycler UNOII (Biometra, Goettingen, Germany) using thin-walled reaction tubes. Two microliters of RNA solution were used for *β-actin*, *MDM2*, *GAGE-1*, *-2* and *FHIT* reverse transcriptase (RT)-PCR. Random hexamers supplied by the manufacturer were used for synthesis of cDNA.

Total RNA extracted from multiple cytological specimens of each patient were pre-screened for *β-actin* RNA by PCR. Final analysis was performed on the samples containing non-degraded *β-actin* RNA. *β-actin*-primers were 5′-CTACAATGAGC-TGCG-TGTGGC-3′ (sense strand) and 5′-CAGGTCCA-GACGCAGGATGGC-3′ (antisense strand) yielding a 240 base pair (bp) product (Albitar et al, 1989). Cycling conditions have been described previously (Schlott et al, 1997).

MDM2 primers used for amplifying the complete C-terminal nucleotide stretch including the zinc finger sequence and the RING finger were 5′-CGACTAGAAGATTATAGCC-3′ (sense primer) and 5′-ATTGGTTGTCTATAGG-3′ (antisense primer). The amplimers yielded a product of 684 bp. The underlying temperature cycling schedule was initiated by a melting step lasting 7 min at 95°C and terminated by a final step at 72°C lasting 10 min. Forty runs were performed. Each run consisted of melting cycle (94°C, 30 s), primer annealing cycle (56°C, 90 s), and extension cycle (72°C, 90 s).

Primer 5′-GACCAAGACGCTACGTAG-3′ (sense strand) and primer 5′-CCATCAGGACCATCTTCA-3′ (antisense strand) were used for amplification of a nucleotide sequence shared by *GAGE-1* and *GAGE-2* RNA. These primers give a 244 bp product. All PCR procedures were performed as described (Van den Eynde et al, 1995).

Primers used for amplification of *FHIT* sequences were 5′-CATCCTGGAAGCTTTGAAGCT-3′ (exon 3, sense primer 1, position –201 to –223), 5′-TCCGTAGTGCTATCTACATCC-3′ (exon 3, sense primer 2, position –142 to –162), and 5′-TCCTCT-GATCTCCAAGAGGC-3′ (exon 9, antisense primer, position 379 to 398). The sequences were selected from published data of Ohta et al (1996). The combination sense primer 1/antisense primer yielded a 599 bp fragment, combination sense primer 2/antisense primer a 561 bp fragment. Following an initial denaturing step at 95°C for 7 min, cDNA was amplified 40 times in three temperature cycles. In each amplification series a 30-s melting cycle at 95°C was followed by a combined annealing cycle for 1 min at 60°C, and an extension cycle for 2 min at 72°C. The final round was completed with a primer extension for 240 s at 72°C. Cycling conditions of the second round PCR differed from the first one in annealing temperature (62°C) and temperature cycle number (30 cycles).

### Gel electrophoresis and cDNA sequencing

For visualization, 5 µl of each PCR assay (final volume 100 µl) was separated on a 3% (w/v) agarose gel containing 0.5 µg ethidium bromide per ml. The gel was registered by means of a CCD camera (Biometra, Goettingen, Germany). *FHIT*-PCR products were separated on 3% low-melting agarose (Biozym, Hameln, Germany) and cut from the gel. The QIAEX II Gel Extraction Kit (QIAGEN, Hilden, Germany) was used to purify the *FHIT* cDNA. The *GAGE-1*, *-2* and *MDM2* amplification products were purified with the QIAquick PCR Purification Kit (QIAGEN, Hilden, Germany).

A total of 200 ng of isolated DNA were labelled with the PRISM Ready Dye Deoxy Terminator Cycle Sequencing Kit (Applied Biosystems, Weiterstadt, Germany) according to the manufacturer's instructions and analysed in an Applied Biosystems DNA sequencer (model 377). Oligonucleotides previously used for amplification of fragments served as sequencing primers.

### RESULTS

In this study, HCC cell lines formerly described by Schlott et al (1997) were used for establishing and optimizing three RT-PCR assays based on the detection of *MDM2*, *GAGE-1*, *-2*, and *FHIT* expression. These tests were ultimately applied on liver tissue and liver cells obtained by fine-needle aspiration.

*© Cancer Research Campaign 1999*

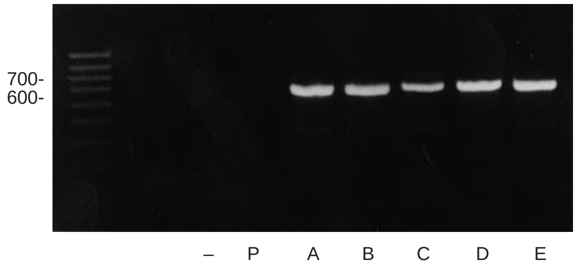

**Figure 1** Detection of *MDM2* mRNA by agarose gel electrophoresis and ethidium bromide staining. RNA from five HCC samples was used for RT-PCR with *MDM2* primers flanking the zinc-finger domain. –, negative control, cell line showing absence of *MDM2* expression; P: buffer control, no RNA in the cDNA synthesis

*MDM2* RNA was detected in all aspirates investigated. Figure 1 is representative for all samples investigated (HCC, FNH, HC). The PCR fragments obtained from the HCC, FNH and HC samples were analysed by automatic sequencing and sequence alignement. *MDM2* point mutations were found exclusively in the *MDM2* transcripts amplified from 3/10 HCC samples (Figure 2). The *MDM2* mutations were located in a zinc-finger domain of MDM2 and could be divided into two groups. Evidence is given for a *MDM2* mutation that has not been described so far. The genetic alteration was classified as a mis-sense mutation replacing the codon CGT by codon AGT. In consequence, the amino acid residue arginine in mature MDM2 might be converted into serine. The other two mutations have been described before and represent a frame-shift mutation inducing a premature stop codon (Schlott et al, 1997).

Expression of the *GAGE-1, -2* genes was found in specimens from six patients with HCC (Figure 3). The PCR fragments did not show any gene mutations or deletions according to the data of automatic sequencing. The other samples (four HCC, ten FNH, ten normal liver specimens) did not contain *GAGE-1, -2* RNA.

Aberrant transcripts were detected by amplifying *FHIT* RNA in seven HCC, two FNH and two HC specimens. Transcript sizes observed in agarose gel ranged from 190 bp to 561 bp (Figure 4). Because of the enhanced sensitivity of the semi-nested assay even low abundance transcripts were detected in the specimens. Five samples (three HCC, two FNH) did not reveal DNA signals corresponding to full length *FHIT* RNA (Figure 4, HCC sample 2). The *FHIT* expression patterns of two HCCs additionally revealed weak DNA bands larger than the expected size of 561 bp representing heteroduplexes formed between *FHIT*-splicing variants.

The nucleotide sequence of the full length *FHIT* cDNA, or the various transcripts found in the samples, was determined by automatic sequencing. None of the *FHIT* mRNAs showed point mutations or nucleotide insertions. In detail, the analysis revealed transcripts lacking exon 5–8, 4–6, 5–7, 5–6, 4–5, and 8 as described in former publications (Negrini et al, 1996; Sozzi et al, 1996; Thiagalingam et al, 1996; Virgilio et al, 1996; Gemma et al, 1997; Hendricks et al, 1997). Most of these transcript types were found in HCC and FNH entities under investigation and negative controls. However, the *FHIT* transcripts lacking exon 5–7 (264 bp) and exon 5–8 (193 bp) occurred in HCC and FNH, samples but not in normal hepatocytes. The nucleotide sequence of both mRNAs is shown in Figure 5. Analysis currently in progress has not yet detected these *FHIT* RNA types in non-neoplastic hepatocytes so far.

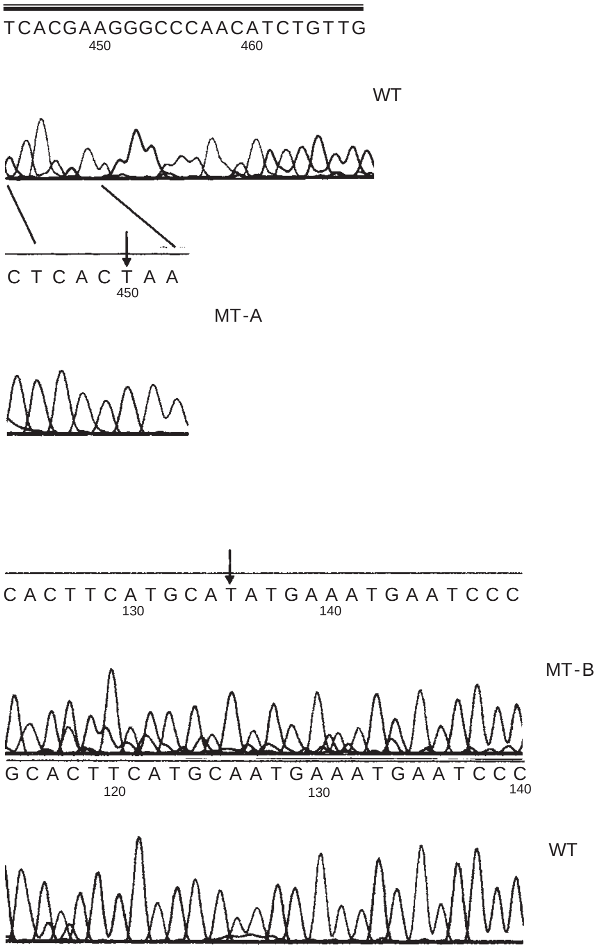

**Figure 2** Sequence analysis of two PCR products amplified from *MDM2* mRNA isolated from HCC. The wild-type (WT) sequences presented were obtained from RNA extracted from a FNH sample. Mutant sequence A (MT-A) obtained from an HCC sample shows mis-sense mutation causing the codon change ACG → ACT (sense strand CGT → AGT). As a result, amino acid *Arg* is replaced by *Ser*. Mutant *MDM2* nucleotide sequence B (MT-B) contains a T-insertion generating a premature stop codon. This frame-shift mutation was detected in two HCC samples. Arrows indicate point mutation/nucleotide insertion

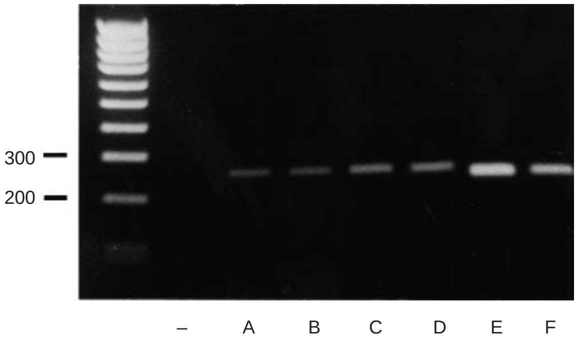

**Figure 3** Identification of 244 bp PCR fragments resulting from the *GAGE-1, -2* genes of HCC samples on 3% agarose gel. The DNA fragment was amplified from six HCC (**A–F**). The sizes of the markers are shown on the left. A FNH sample served as negative control (–)

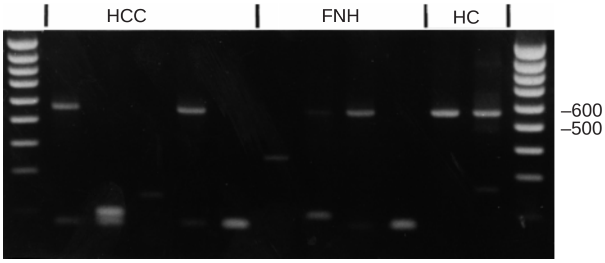

**Figure 4** Analysis of *FHIT* expression in a panel of liver aspirates on 3% agarose gel. Each lane shows the PCR fragments produced by RT-PCR and semi-nested PCR. The lanes contain normal (561 bp) and various aberrant *FHIT* transcripts (195 bp, 200 bp, 264 bp, 290 bp, 350 bp, 490 bp) detected in HCC (lanes 1–5), FNH (lanes 6–9), and two HC (lanes 10, 11) serving as negative controls. The sizes of the markers are shown on the right

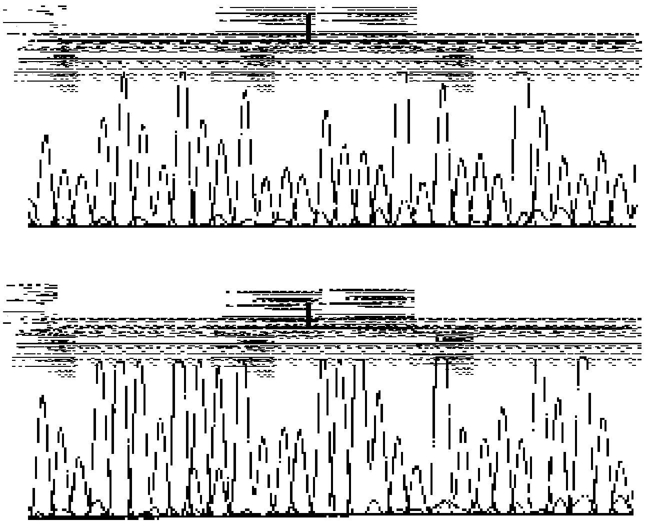

**Figure 5** Automatic sequencing of two aberrant *FHIT* transcripts. Fragment A (264 bp length) lacked exons 5–7. In fragment B (195 bp length) exons 5–8 were deleted. Both types of transcript were found in HCC and FNH, but not in hepatocytes.

**Table 1** Expression analysis of *FHIT*, *GAGE-1*, *-2*, and *MDM2* in the investigated HCC fine-needle aspirates.

| HCC sample No. | Full length *FHIT* RNA | Aberrant *FHIT* RNA | *FHIT* transcript, exons deleted | *GAGE-1*, *-2* RNA | *MDM2* mutation |
|---|---|---|---|---|---|
| 1 | + | + | 5–8 | + | – |
| 2 | – | + | 5–8, 4–6, 5–6 | – | + (I) |
| 3 | – | + | 5–8, 5–7 | – | + (C) |
| 4 | + | + | 5–8, 4–5, 8 | + | – |
| 5 | + | + | 5–6 | – | – |
| 6 | + | + | 5–8 | + | – |
| 7 | – | + | 5–8 | + | – |
| 8 | + | + | 4–5, 8 | + | – |
| 9 | + | + | 5–8, 4–6, 4–5 | + | – |
| 10 | + | + | 4–6 | – | + (I) |

I, T-insertion; C, Arg → Ser conversion

An overview on the expression pattern of HCC aspirates is demonstrated in Table 1.

### DISCUSSION

From a clinical point of view, fine-needle aspiration biopsy is an important adjunct for diagnosing benign and malignant lesions of the liver. The cytodiagnostic criteria used for HCC are increased nucleocytoplasmic (N/C) ratio, trabecular pattern, atypical naked nuclei, bile production by malignant hepatocytes, multinucleated tumour cells and absence of bile duct epithelium (Ducatman, 1992). However, morphological changes associated with inflammatory and proliferative processes of the liver may often mimic malignancy in fine-needle aspirates. In particular, the differentiation between well-differentiated HCC and benign reactive conditions (i.e. FNH) can be very difficult in cytodiagnosis (Huebscher and Young, 1993).

In search for genetic markers associated with malignant liver neoplasms, benign and malignant cells of human liver were evaluated for genetic alterations concerning the oncogene *MDM2*, the *GAGE-1*, *-2* genes, and the candiate tumour suppressor gene *FHIT* in the present study. Accordingly, sensitive nested RT-PCR and automatic laser scanning system were combined to characterize the expression and mutation of the target genes. Of note, other genetic techniques formerly used for this purpose, e.g. Southern blot hybridization and LOH analysis were not taken into consideration since they are difficult to perform on cytological specimens containing heterogeneous cell populations.

The *MDM2* gene of many solid tumours reveals characteristic genetic alterations; among these, the most frequently recognized is gene amplification which is referred to as a major factor of *MDM2* overexpression and an indicator of malignancy. Concerning liver neoplasms, amplification of the *MDM2* should be a rare event since studies on hepatoblastoma cells showed non-amplified *MDM2* genes in all samples analysed (Ohnishi et al, 1996). However, different alterations of *MDM2* were detected recently in two HCC cell lines and further tumour entities, e.g. point mutations in a putative zinc-finger motif of *MDM2* (Schlott et al, 1997). In the present work, the respective assays were transfered to a larger pool of aspirates containing malignant or benign liver cells. Expression of the *MDM2* gene was detected in all HCC, FNH and HC samples investigated. Interestingly, *MDM2* point mutation was observed in HCC sample leading to conversion from Arg to Ser or to the loss of the three zinc-finger motifs. The functional consequences of the amino acid substitution located adjacent to zinc-finger motif 1 are speculative since NMR and crytallographical analysis on this phylogenetically conserved domain is lacking. However, the effects of the T-insertion, which was formerly detected in two HCC cell lines (Schlott et al, 1997), should be discussed here in view of current publications. Accordingly, it has been shown recently for MDM2 protein that the C-terminal RING domain is involved in the repression of the *cyclin-A* gene which controls the transition from G1 to S phase. When transfecting cells with MDM2 RNA whose zinc-finger domain was deleted, high levels of cyclin-A (Leveillard and Wasylyk, 1997) often observed in MDM2 transformed cells, were noted. Moreover, *MDM2* transcripts lacking the complete 3′-end with the three zinc-finger motifs naturally occur in tumour cells. These transcripts are able to transform cultivated mouse fibroblasts (Sigalas et al, 1996). Thus, it might be speculated that *MDM2* frame-shift mutations detected

 © Cancer Research Campaign 1999

in HCC are associated with synthesis of truncated MDM2 proteins contributing to malignancy. In vitro transfection assays on mouse hepatocytes might help to prove this hypothesis.

Expression of one of the proteins belonging to the family of tumour-associated antigens, i.e. MAGE-1, has already been reported for HCC (Yamashita et al, 1996); however, neoplasms of the liver have apparently not yet been analysed for the expression of the *GAGE-1*, *-2* genes. In the present study, *GAGE-1*, *-2* RNA was detected in 60% of HCC specimens, although some of the fine-needle aspirates have been stored for years. Interestingly, the data of Yamashita and co-workers (1996) revealed a comparable high percentage of *MAGE-1* expression (75%) in fresh HCC samples derived from HCC cell lines and biopsies, respectively. The results of both studies favour the application of RNA-PCR assays on various kinds of material.

It is noteworthy that *MDM2* mutations were only found in HCC cells showing absence of the *GAGE-1*, *-2* expression. It suggests that synthesis of intact MDM2 proteins in HCC is associated with up-regulation of *GAGE-1*, *-2* genes. However, this fact should be confirmed by further in vitro experiments on cultivated HCC cells in order to prove any physiological connection between both markers.

*FHIT* was selected for analysis, since former data indicated that aberrant *FHIT* transcripts may be potential indicators of malignancy. The present data indicate expression of aberrant *FHIT* transcripts in malignant as well as in non-malignant liver cells of archival fine-needle aspirates. This phenomenon is possibly due to the applying of semi-nested-PCR technique which favoured amplification of *FHIT* transcripts produced in small amounts. While this work was in progress, Chen et al (1998), also detected aberrant *FHIT* transcripts in fresh non-tumorous liver tissue. Similar results were obtained by other groups in premalignant and malignant lesions of bronchi, liver and brain (Fong et al, 1997; Panagopoulos et al, 1997). Taken together, these observations from different groups working independently still leave the question open whether it is possible to relate the detection of *FHIT*-splicing variants to malignant transformation.

Concerning liver cells, our data are not conclusive whether a characteristic *FHIT* expression pattern exists which might help to differentiate between non-neoplastic and neoplastic liver cells. This is due to the fact that two types of *FHIT* transcript lacking exons 5–7 and exons 5–8 were detected in HCC and FNH, but not in the negative controls. Thus, it can be argued that both transcripts might play a role in proliferation of malignant and non-malignant liver cells, but further experiments on a larger collection of hepatic samples are necessary to support this hypothesis. Interestingly, Fong and co-workers recently detected the transcript lacking exons 5–8 in Epstein–Barr virus-infected cell line and supposed it to be the result of transformation (Fong et al, 1997).

Other typical genetic alterations were not detectable in malignant and non-malignant liver cell *FHIT* RNA. Mutations, for example, which would be reliable indicators for inactivation of the putative *FHIT* tumour suppressor, could not be detected in either the histidine triad motif His-Xaa-HisXaa-His of exon 8, a highly conserved region possibly involved in binding of zinc ions (Lima et al, 1996), or in other regions of *FHIT* amplification products. These results are in accordance with the data of Sozzi et al (1996) and Ohta et al (1996), who found *FHIT* mutations in coding regions to be rare events.

In summary, the *FHIT* assay did not allow differentiation between malignant hepatocytes, benign proliferating hepatocytes and normal hepatocytes tested in this study. Since formation of aberrant *FHIT* RNA appears to be a common feature of benign, non-neoplastic hepatocytes, the role of *FHIT* transcripts in hepatocellular transformation is elusive. In contrast, *MDM2* mutations and *GAGE-1*, *-2* expression were only present in HCC. Therefore, it might be of value to investigate whether the two assays can be used in routine cytodiagnosis of liver lesions.

Taking all demonstrated facts together, one can conclude that combining the *GAGE-1*, *-2* PCR assay with other PCR assays based on the detection of other tumour rejection antigens, such as MAGE-1 and MAGE-3, may further optimize genetic detection of HCC in cytological material. Studies are currently under progress to test this hypothesis.

### REFERENCES

Albitar M, Peschle C and Liebhaber SA (1989) Theta, zeta and epsilon globin messenger RNAs are expressed in adults. *Blood* **2**: 629–637

Boddy MN, Freemont PS and Borden KLB (1994) The p53-associated protein MDM2 contains a newly characterized zinc-finger domain called the RING finger. *Trends Biochem Sci* **19**: 188–189

Böel P, Wildmann C, Sensi ML, Brasseur R, Renauld JC, Coulie P, Boon T and Van der Bruggen P (1995) *BAGE*, a new gene encoding an antigen recognized on human melanomas by cytolytic T-lymphocytes. *Immunity* **2**: 167–175

Bueso-Ramos CE, Yang Y, DeLeon E, McCown P, Stass SA and Albithar M (1993) The human *MDM2*-oncogene is overexpressed in leukemias. *Blood* **82**: 2617–2623

Cohen AJ, Li FP, Berg S, Marchetto DJ, Tsai S, Jacobs SC and Brown RS (1979) Hereditary renal cell carcinoma associated with a chromosomal translocation. *N Engl J Med* **301**: 592–595

Chen YJ, Chen PH and Chang JG (1998) Aberrant FHIT transcripts in hepatocellular carcinomas. *Br J Cancer* **77**: 417–420

Ducatman BS (1992) Fine needle aspiration of the liver and pancreas. In *Atlas of Diagnostic Cytopathology*, Mitchell J (ed), pp. 319–320. WB Saunders: Philadelphia

Fakharzadeh SS, Trusko SP and George DL (1991) Tumorigenic potential associated with enhanced expression of a gene that is amplified in mouse tumor cell line. *EMBO J* **10**: 1565–1569

Finlay CA (1993) The *MDM2* oncogene can overcome wild-type p53 suppression of transformed cell growth. *Mol Cell Biol* **13**: 301–306

Fong KM, Biesterveld EJ, Virmani A, Wistuba I, Sekido Y, Bader SA, Ahmadian M, Ong ST, Rassool FV, Zimmermann PV, Giaccone G, Gazdar AF and Minna JD (1997) *FHIT* and FRA3B 3p14.2 allele loss are common in lung cancer and pre-neoplastic bronchial lesions and are asociated with cancer-related *FHIT* cDNA splicing aberrations. *Cancer Res* **57**: 2256–2267

Gaugler B, Van den Eynde B, Van der Bruggen P, Romero P, Gaforio JJ, DePlaen E, Lethe B, Brasseur F and Boon T (1994) Human gene *MAGE*-3 codes for an antigen recognized on a melanoma by autologous cytolytic T lymphocytes. *Exp Med* **179**: 921–930

Gemma A, Hagiwara K, Ke Y, Burke LM, Khan MA, Nagashima M, Bennett WP and Harris CC (1997) *FHIT* mutations in human primary gastric cancer. *Cancer Res* **57**: 1435–1437

Hendricks DT, Taylor R, Reed M and Birrer J (1997) *FHIT* gene expression in human ovarian, endometrial and cervical cancer cell lines. *Cancer Res* **57**: 2112–2115

Huebscher SG and Young JA (1993) Liver. In *Fine Needle Aspiration Cytopathology*, Young JA (ed), pp. 141–142. Blackwell Scientific Publications: Oxford

Kastury K, Baffa R, Druck T, Ohta M, Cotticelli MG, Inoue H, Negrini M, Rugge M, Huang D, Croce CM, Palazzo J and Huebner K (1996) Potential gastrointestinal tumor suppressor locus at the 3p 14.2 FRA3B site identified by homozygous deletions in tumor cell lines. *Cancer Res* **56**: 978–983

LaForgia S, Morse B, Levy J, Barnea G, Cannizzaro LA, Li F, Nowell PC, Boghosian-Sell L, Glick J, Weston A, Harris CC, Drabkin H, Patterson D, Croce CM, Schlessinger J and Huebner K (1991) Receptor protein-tyrosine phosphatase γ is a candidate tumor suppressor gene at human chromosome region 3p21. *Proc Natl Acad Sci USA* **88**: 5036–5040

LeBeau MM and Rowley JD (1984) Heritable fragile sites in cancer. *Nature* **308**: 607–608

Leveillard T and Wasylyk B (1997) The MDM2 C-terminal region binds to TAFII250 and is required for MDM2 regulation of the *cyclin-A* promoter. *J Biol Chem* **272**: 30651–30661

© Cancer Research Campaign 1999 

Lima CD, Klein MG, Weinstein B and Hendrickson WA (1996) Three-dimensional structure of human protein kinase C interacting protein, a member of the HIT family of proteins. *Proc Natl Acad Sci USA* **93**: 5357–5362
Momand J, Zambetti GP, Olson DC, George D and Levine AJ (1992) The *MDM2* oncogene product forms a complex with the p53 protein and inhibits p53-mediated transactivation. *Cell* **69**: 1237–1245
Negrini M, Monaco C, Vorechovsky I, Ohta M, Druck T, Baffa R, Huebner K and Croce CM (1996) The *FHIT* gene at 3p14.2 is abnormal in breast carcinomas. *Cancer Res* **56**: 3173–3179
Ohnishi H, Kawamura M, Hanada R, Kaneko Y, Tsunoda Y, Hongo T, Bessho F, Yokomori K and Hayashi Y (1996) Infrequent mutations of the *TP53* gene and no amplification of the *MDM2* gene in hepatoblastoma. *Genes Chromosomes Cancer* **15**: 187–190
Ohta M, Inoue H, Cotticelli MG, Kastury K, Baffa R, Palazzo J, Siprashvili Z, Mori M, McCue P, Druck T, Croce CM and Huebner K (1996) The *FHIT* gene spanning the chromosome 3p14.2 fragile site and renal carcinoma-associated t(3;8) breakpoint, is abnormal in digestive tract cancers. *Cell* **84**: 587–597
Oliner JD, Kinzler KW, Meltzer PS, George DL and Vogelstein B (1992) Amplification of a gene encoding a p53 associated protein in human sarcomas. *Nature* **358**: 80–83
Panagopoulos I, Thelin S, Mertens F, Mitelman F and Aman P (1997) Variable *FHIT* transcripts in non-neoplastic tissues. *Genes Chromosom Cancer* **19**: 215–219
Russo V, Dlerba P, Ricci A, Bonazzi C, Leone BE, Mangioni C, Allavena P, Bordignon C and Traversari C (1996) *MAGE*, *BAGE* and *GAGE* gene expression in fresh epithelial ovarian carcinomas. *Int J Cancer* **67**: 457–460
Schlott T, Reimer S, Jahns A, Ohlenbusch A, Ruschenburg I, Nagel H and Droese M (1997) Point mutations and nucleotide insertions in the MDM2 zinc-finger structure of human tumours. *J Pathol* **182**: 54–61
Sigalas I, Calvert H, Anderson JJ, Neal DE and Lunec J (1996) Alternatively spliced *MDM2* transcripts with loss of p53 binding domain sequences: transforming ability and frequent detection in human cancer. *Nat Med* **2**: 912–917
Sozzi G, Veronese ML, Negrini M, Baffa R, Cotticelli MG, Inoue H, Tornielli S, Pilotti S, DeGregorio L, Pastorino U, Pierotti MA, Ohta M, Huebner K and Croce CM (1996) The *FHIT* gene at 3p14.2 is abnormal in lung cancer. *Cell* **85**: 17–26
Sozzi G, Sard L, DeGregorio L, Marchetti A, Musso K, Buttitta F, Tornielli S, Pellegrini S, Veronese ML, Manenti G, Incarbone M, Chella A, Angeletti CA, Pastorino U, Huebner K, Bevilaqua G, Pilotti S, Croce CM and Pierotti MA (1997) Association between cigarette smoking and *FHIT* gene alterations in lung cancer. *Cancer Res* **57**: 2121–2123
Thiagalingam S, Lisitsyn NA, Hamaguchi M, Wigler MH, Willson JKV, Markowitz SD, Leach FS, Kinzler KW and Vogelstein B (1996) Evaluation of the *FHIT* gene in colorectal cancers. *Cancer Res* **56**: 2936–2939
Van den Eynde B, Peeters O, De Backer O, Gaugler B, Lucas S and Boon T (1995) A new family of genes coding for an antigen recognized by autologous cytoloytic T lymphocytes on a human melanoma. *J Exp Med* **182**: 689–698
Van der Bruggen P, Traversari C, Chomez P, Lurquin C, De Plaen E, Van den Eynde B, Knuth A and Boon T (1991) A gene encoding an antigen recognized by cytolytic T lymphocytes on a human melanoma. *Science* **254**: 1643–1647
Virgilio L, Shuster M, Gollin SM, Veronese ML, Ohta M, Huebner K and Croce CM (1996) *FHIT* gene alterations in head and neck squamous cell carcinomas. *Proc Natl Acad Sci USA* **93**: 9770–9775
Wang N and Perkins KL (1984) Involvement of band 3p14 in t(3;8) hereditary renal carcinoma. *Cancer Genet Cytogenet* **11**: 479–481
Yamashita N, Ishibashi H, Hayashida K, Kudo J, Takenaka K, Itho K and Niho Y (1996) High frequency of the *MAGE-1* gene expression in hepatocellular carcinoma. *Hepatology* **24**: 1437–1440

*© Cancer Research Campaign 1999*
